# Association between real-time polymerase chain reaction cycle threshold value and clinical presentation in children with *Bordetella pertussis*

**DOI:** 10.1186/s13052-024-01753-3

**Published:** 2024-09-11

**Authors:** Wen Li, Huaping Wang, Shu Teng, Yalin Sun, Qi Jia, Zhenghong Qi, Lingbo Wang, Zhangnv Yang, Shiyong Zhao

**Affiliations:** 1 Department of Pediatric Infectious Diseases, Hangzhou Children’s Hospital, 195 Wenhui Road, Hangzhou, 310014 Zhejiang China; 2 Department of Microbiology, Zhejiang Provincial Center for Disease Control and Prevention, Hangzhou, China

**Keywords:** Pertussis, Cycle threshold, Polymerase chain reaction

## Abstract

**Background:**

The cycle threshold (Ct) value is inversely proportional to the number of copies of the target region in a sample, suggesting that a low Ct value indicates a high pathogen load. The relationship between Ct value and clinical presentation in children with pertussis is not well-defined.

**Methods:**

We investigated the relationships between the Ct value of nasopharyngeal samples positive for *Bordetella pertussis* deoxyribonucleic acid via real-time polymerase chain reaction (PCR), collected from children on admission and their adult family members between May 2022 and March 2024 at Hangzhou Children’s Hospital, China. The study focused on the correlation between Ct value and clinical presentation in children with pertussis.

**Results:**

The Ct value was positively correlated with age (*r* = 0.362, *P* = 0.001). The mean Ct value for children with pertussis was 28.0 (range: 22.0–32.0), which was lower than the 32.0 (range: 30.0–34.0) observed in adults. Ct value was inversely correlated with length of stay, an indicator of disease severity (*r* = -0.356, *P* = 0.001). Logistic regression analyses revealed that both Ct value (OR: 0.891, 95% CI: 0.799–0.993, *P* = 0.036) and white blood cell count (OR: 1.127, 95% CI: 1.005–1.263, *P* = 0.040) were independently associated with severity of pertussis.

**Conclusions:**

Real-time PCR Ct values at initial diagnosis for pertussis may potentially predict severe disease outcomes in children.

## Background

Pertussis (whooping cough) is a highly contagious acute respiratory infection caused by *Bordetella pertussis* [1].Effective infant immunization programs have significantly decreased the incidence of pertussis. Nevertheless, a resurgence of pertussis over the past two decades, observed in both developed and developing countries, indicates that it remains a public health concern [2–4].China has seen an unusual increase in pertussis cases over the past two years, coinciding with shifts in strategies against Corona Virus Disease 2019 (COVID-19). This resurgence highlights the ongoing public health challenge in China, with a record 15,275 cases reported in January 2024 [5]. Globally, pertussis remains a significant concern; Yeung et al., using World Health Organization data, reported approximately 24.1 million cases and 160,700 deaths in children under 5 years old in 2014 [6]. This resurgence might contribute to the replacement of acellular vaccines, variations in pertussis pathogens and epidemiological changes against vaccine pressure, increased public awareness of this disease, improved diagnostic tests, and so on [7]. Traditionally, bacterial culture has been considered the gold standard for diagnosing pertussis, but its low sensitivity and lengthy process limit its diagnostic utility. In recent years, the efficacy of real-time polymerase chain reaction (RT-PCR) for diagnosing whooping cough has gained wide recognition [8–10]. The cycle threshold (Ct) value, which indicates the number of amplification cycles required for the amplicon to exceed a basal fluorescence threshold level, is a critical measure in this process. A lower Ct value suggests the presence of significant deoxyribonucleic acid (DNA) levels in an earlier amplification cycle, indicating a higher bacterial DNA concentration.

Research on the relationship between clinical presentation or outcomes and the Ct value for influenza, human rhinovirus, and respiratory syncytial virus has produced conflicting conclusions [11], and the relationship between Ct value and clinical presentation in children with pertussis is still unclear. Consequently, we performed real-time PCR diagnostics for pertussis and assessed the relationships between disease severity, other clinical variables, and Ct value.

## Methods

### Study design and patients

This observational single-center study was conducted between May 2022 and March 2024 at Hangzhou Children’s Hospital. It included hospital admissions for children diagnosed with pertussis via positive *Bordetella pertussis* PCR. Clinical data were extracted from the patients’ medical records, capturing details such as sex, age, symptoms, immunization status, past medical history, comorbidities, duration of hospital stay, time to diagnosis, and laboratory results. These results included the Ct value, chest radiograph or computed tomography, white blood cell (WBC) count, and nucleic acids of common respiratory pathogens.

### Definition

We selected inspiratory rooster-like roaring, vomiting after coughing, recurrent apnea, paroxysmal cyanosis, and fever as the primary symptoms observed. The length of stay (LOS), dichotomized by a median split (< 8 days; ≥ 8 days), was chosen as the outcome measure of disease severity, given its availability in medical records and clinical relevance.

Common viral causes of respiratory tract infections include influenza virus (IFV), respiratory syncytial virus (RSV), human metapneumovirus (HMPV), parainfluenza virus (PIV), human rhinovirus (HRV), human adenovirus (HAdV), human coronavirus (HCoV), human bocavirus (HBoV), Mycoplasma pneumoniae (MP), and Chlamydia. Not all patients were simultaneously detected with all these pathogens.

In China, children receive the DTaP vaccine as primary immunization at 3, 4, and 5 months, followed by a booster dose at 18–24 months [12]. Based on their vaccination status, patients were classified as “completely vaccinated,” “incompletely vaccinated,” or “unvaccinated.” Furthermore, the children were categorized into five age groups at the time of diagnosis: under 3 months, 3–6 months, 7–12 months, 1–5 years, and over 5 years.

### Sample collection

Upon admission, nasopharyngeal swab samples were promptly collected from both patients and close family members for real-time PCR and bacterial culture. These samples were stored at -20 °C until testing. The laboratory at the Institute of Infectious Diseases, Zhejiang Center for Disease Control and Prevention, performed these tests.

### Real-time PCR

Real-time PCR targeting IS481 and ptxP was conducted on all suspected *B*. *pertussis* isolates using the Applied Biosystems 7500 Fast Real-Time PCR System (Thermo Fisher Scientific, Waltham, MA, US). The procedure began by adding 600 µL of autoclaved normal saline to the sampling tube of the collected nasopharyngeal swab, followed by vortexing and shaking for about 15 s. Subsequently, 200 µL of the solution from the sampling tube was transferred to an EP tube. *B*. *pertussis* DNA was extracted using the QIAamp DNA Mini Kit (QIAGEN, Valencia, CA, USA) according to the manufacturer’s instructions. PCR primers were synthesized based on the Monitoring Technical Manual of the National Pathogen Identification Network Laboratory, with the sequence detailed in the accompanying table. PCR conditions were as follows: an initial hold at 50 °C for 2 min and 95 °C for 3 min (1 cycle), followed by 40 cycles of 95 °C for 5 s and 55 °C for 1 min. Positive and negative controls were tested concurrently with the samples to validate each assay. The Ct value, indicative of the bacterial DNA load in the nasopharynx, was calculated concurrently with the sample testing. A Ct value below 35 and an amplification curve showing a plateau or near-plateau indicate a positive result; otherwise, it is negative.

### Statistical analyses

Statistical analyses were performed using Statistical Package for the Social Sciences (version 23.0; IBM, Armonk, NY, USA). Skewed data are presented as the median (interquartile range, IQR) and were analyzed using the Mann-Whitney U test. The correlations of Ct value with numerical variables were assessed using the Spearman correlation test. Logistic regression analysis was conducted on variables that were correlated with severe disease or that could potentially affect disease severity. P-values < 0.05 were considered statistically significant.

## Results

### Baseline patient characteristics

A total of 110 nasopharyngeal swab samples from children were submitted to the diagnostic laboratory. During the study period, 84 respiratory samples tested positive for *Bordetella pertussis* by PCR. The baseline characteristics of these 84 patients are detailed in Table 1. The median age of the patients was 36.0 months (IQR: 3.3–68.0 months). Only two patients had not received antibiotics before admission.

**Table 1:** Baseline characteristics of the study population

Characteristics	No. of subjects (*n* = 84)
Sex	
Male Female	41(48.8%) 43(51.2%)
Age	
< 3 months	
3‒6 months 7‒12 months 1‒5 years > 5 years	16(19.1%) 17(20.2%) 4(4.8%) 19(22.6%) 28(33.3%)
Immunization status	
Completely vaccinated Incompletely vaccinated Unvaccinated	49(58.3%) 9(10.7%) 26(31.0%)

### Ct value and vaccination status

The Ct value was significantly lower for unvaccinated patients (24.0, IQR: 20.0–30.5, *n* = 26) compared to those who were vaccinated or incompletely vaccinated (30.0, IQR: 24.8–32.0, *n* = 58) (*P* = 0.011).

### Ct value and clinical presentation

#### Symptoms

In our study, the most frequent symptom in children with pertussis was post-tussive vomiting (69.0%), followed by inspiratory cockcrow-like roaring (57.1%), paroxysmal cyanosis (29.8%), fever (33.3%), and recurrent apnea (4.8%). The Ct value showed a weak negative correlation with the number of symptoms (*r* = -0.264, *P* = 0.015, *n* = 84), indicating that children with lower Ct value tended to experience more symptoms. Associations between Ct value and clinical characteristics are detailed in Table 2.

**Table 2:** Associations between ct value and clinical characteristics

Variable	*n*(%)	Ct value Median (Q1,Q3)	Statistic	*P* value
Sex				
Male Female	41(48.8) 43(51.2)	30.0(22.5,32.0) 28.0(22.0,32.0)	0.036	0.971
Age				
≤ 1 year > 1year	37(44.1) 47(55.9)	24.0(20.0,29.0) 30.0(28.0,33.0)	−3.734	< 0.001*
Immunization status				
Unvaccinated Completely or Incompletely vaccinated	26(31.0) 58(69.0)	24.0(20.0,30.5) 30.0(24.8,32.0)	−2.534	0.011*
Co-infection				
Yes No	36(42.9) 48(57.1)	31.5(27.3,33.8) 26.0(20.5,30.0)	−3.442	0.001*
Pneumonia				
Yes No	42(50.0) 42(50.0)	28.0(22.0,31.3) 30.0(23.5,32.0)	−1.005	0.315

*Statistically significant

### Clinical severity

The median LOS in the hospital was 8 days (IQR: 6.0–10.0 days). The Ct value was inversely correlated with LOS (*r* = -0.356, *P* = 0.001, *n* = 84), indicating that a lower Ct value is associated with longer hospital stays. In this study, LOS dichotomized as less than 8 days or 8 days and above, served as a marker for disease severity. Logistic regression analyses showed that both WBC count (OR: 1.127, 95% CI: 1.005–1.263, *P* = 0.040) and Ct value (OR: 0.891, 95% CI: 0.799–0.993, *P* = 0.040) were significantly associated with severe disease (Table 3).

**Table 3:** Logistic regression analysis of factors associated with severe disease (length of stay)

Variables	B	Standard Error	Wald	*P* value	OR	95% CI
Constant	2.562	1.965	1.701	0.192	12.967	
WBC counts	0.119	0.058	4.207	0.040*	1.127	1.005 ~ 1.263
Age	-0.014	0.008	3.240	0.072	0.986	0.971 ~ 1.001
Ct value	-0.116	0.055	4.375	0.036*	0.891	0.799 ~ 0.993
Vaccination (received at least 1 dose)	0.117	0.785	0.022	0.882	1.124	0.241 ~ 5.234

*Statistically significant

### Co-infection

Co-infections were identified in 36 (42.9%) of the 84 pertussis cases studied while testing for other respiratory pathogens. The most frequently detected co-pathogens were HRV with 14 cases, followed by MP with 8 cases, IFV and HAdV each with 5 cases, RSV with 4 cases, PIV with 3 cases, and HCoV with 1 case. Notably, six patients with pertussis were simultaneously infected with two other pathogens. The Ct value of co-infected patients was significantly higher than that of non-co-infected patients (*P* = 0.001).

### Pneumonia

All patients underwent chest X-rays or computed tomography, and 42 (50.0%) were diagnosed with pneumonia. However, no significant difference in Ct value was observed between the pneumonia group and the non-pneumonia group (*P* = 0.315).

### Other clinical parameters

The Ct value was positively correlated with age (*r* = 0.362, *P* = 0.001, *n* = 84). Infants with pertussis had significantly lower Ct values (24.0, IQR: 20.0–29.0, *n* = 37) compared to older children (30.0, IQR: 28.0–33.0, *n* = 47) (*P* < 0.001). Additionally, the Ct value was inversely correlated with WBC count (*r* = -0.416, *P* < 0.001, *n* = 84), with 54.8% (46/84) of patients exhibiting elevated WBC levels. The median time from the onset of the first symptom to diagnosis was 17.0 days (IQR: 14.0–23.8 days). There was also a correlation between the Ct value and the time from initial symptom onset to diagnosis (*r* = 0.248, *P* = 0.023, *n* = 84).

### Ct value in adults and children

**Fig. 1:**
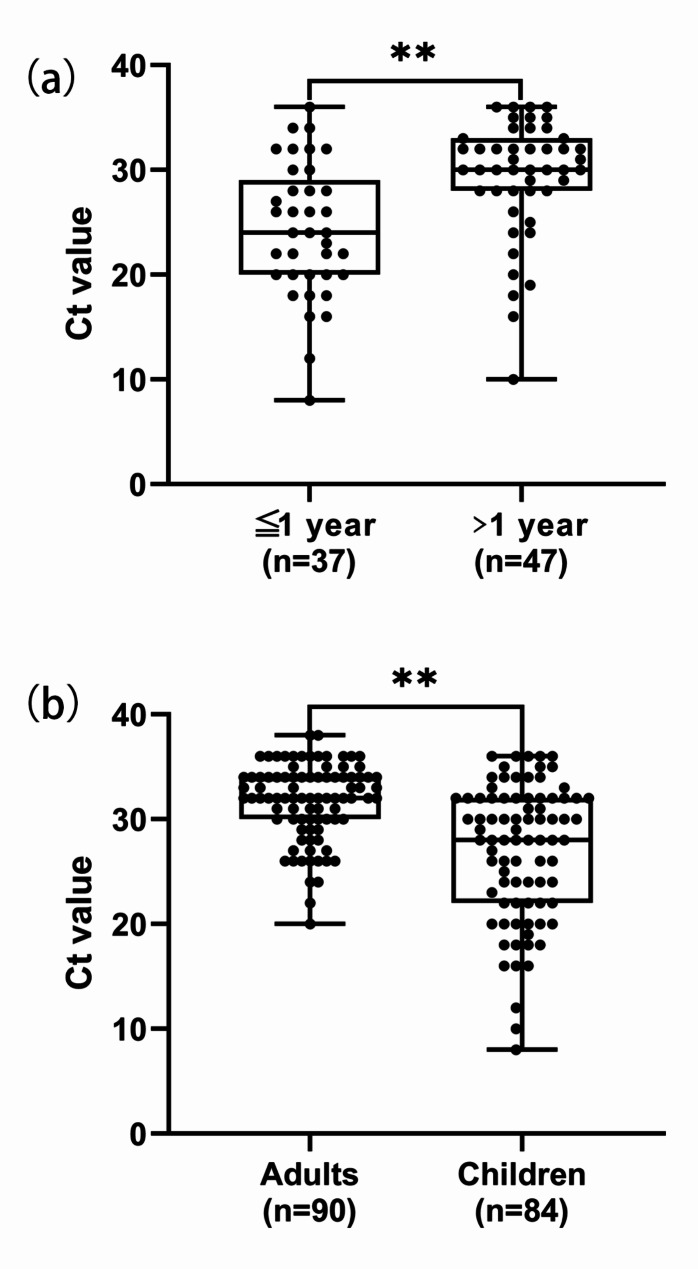
Comparison of Ct value in nasopharyngeal swabs among children and adults with pertussis. (**A**) Infants (*n* = 37) versus older children (*n* = 47). (**B**) Adults (*n* = 90) versus children (infant and older children, *n* = 84)

A total of 119 adult nasopharyngeal swabs were tested, with 90 (75.6%) testing positive. Figure 1 displays the Ct values of PCR-positive samples from 84 children and 90 adults. The mean Ct value for children with pertussis was 28.0 (range: 22.0–32.0), which was lower than the mean Ct value of 32.0 (range: 30.0–34.0) for adults with pertussis. A statistically significant difference in Ct values between children and adults was observed (*P* < 0.001).

## Discussion

Previously, PCR testing for pertussis was limited to a few tertiary hospitals in China. However, with the development and promotion of domestic pertussis kits, their use has expanded due to the associated speed and accuracy. Despite this, there are still relatively few studies examining the relationship between pertussis DNA levels and clinical characteristics. Our findings indicate that low real-time PCR Ct values at diagnosis are associated with severe pertussis, suggesting that Ct values could serve as a predictive indicator of disease severity of pertussis in children.

Numerous studies have explored factors linked with severe pertussis, such as increased WBC count [13], young age [14], unvaccinated [15] and pulmonary hypertension [16]. DeVincenzo et al. investigated the relationship between pertussis DNA load, age, immunization status, and other variables with disease severity, as gauged by LOS. They discovered that a higher bacterial load was associated with increased LOS, indicative of more severe disease [17]. Similarly, Brotons et al. used hospitalization as a marker of severity and found that the combination of bacterial load with other prognostic factors, such as number of symptoms, immunization status, age, provided a reliable prediction for hospitalization [18]. Aligning with these findings, a study conducted in Vietnam on newborns and infants with pertussis also identified a correlation between Ct value and the severity of clinical disease [19].

In a previous study conducted in the United States, pertussis vaccination was found to reduce both the severity and duration of pertussis [20]. Consistent with DeVincenzo et al. [17], our findings indicate that lower Ct values were observed in unvaccinated patients. Notably, 43.2% of the cases under one year old in our study were too young to be vaccinated, as they were under three months of age. This points to a potential gap in the current immunization program, which may not provide adequate protection for the youngest infants. Supporting this, Foxwell et al. reported that advancing the initial dose of the pertussis vaccine from 8 weeks to 6 weeks significantly reduced pertussis incidence, hospitalization rate, and hospital bed-days [21]. Exploring the feasibility of administering the pertussis vaccine to Chinese infants at 2 months, or even as early as 6 weeks of age, could be beneficial in future efforts to enhance disease prevention.

We demonstrated that Ct values are inversely correlated with WBC count. A lower Ct level, which indicates a higher bacterial load, results in increased production of pertussis toxin (PT). PT is associated with an abnormally elevated WBC count [22]. Consistent with previous research, elevated peripheral blood leukocyte counts in pertussis have been positively correlated with disease severity in earlier studies [23, 24].

Numerous studies have identified family members as the primary source of pertussis infection in children [25, 26]. In our study, *Bordetella pertussis* was detected via RT-PCR in family members who had close contact with diagnosed children, including 57.8% (52/90) of adults with cough symptoms and 42.2% (38/90) who were asymptomatic. Often, coughing among these close contacts occurred before the onset of cough in children.Notably, 77.4% (65/84) of children with pertussis had at least one family member also diagnosed with the disease, highlighting the crucial role of adults in transmitting pertussis within households. Generally, adults with pertussis exhibit less typical symptoms than children [27], which could contribute to underdiagnoses in this group. Consistent with previous research, we found a positive correlation between Ct value and age [27, 28]. Our findings suggest that adults, especially compared to infants, tend to have higher Ct levels in their nasopharyngeal secretions, which may correlate with more atypical and milder symptoms. This phenomenon could justify the administration of additional vaccine doses to adults and older children to mitigate the spread of pertussis.

In our study, a significant proportion (42.9% or 36/84) of children with pertussis were coinfected with other pathogens. This finding aligns with those of Frassanito et al., who reported that 47.2% of infants hospitalized with pertussis in Italy were usually coinfected with respiratory viruses [29]. Similarly, studies from northern China (Beijing) and southern China (Suzhou) reported pertussis co-infection rates of 37.9% and 40.1%, respectively [30, 31]. Notably, our study’s co-infections did not include other bacteria, likely leading to an underestimation of the true prevalence of co-infections in pertussis cases. HRV was the most commonly identified co-infecting virus, present in 38.9% (14/36) of co-infected patients, a finding consistent with the literature [29, 30]. Other studies have also highlighted HAdV or PIV as prevalent co-infecting viruses [31, 32]. Variations in social demographics and the year of study may influence the documented rates of co-infection. Interestingly, the Ct value in co-infected patients was significantly higher than that in those without co-infections. This suggests that the presence of other pathogens might inhibit the growth of pertussis bacteria, impacting the bacterial load reflected in the Ct values.

The present study has several limitations. First, its single-center design may limit the generalizability of the findings. Second, there was no follow-up of patients; many were reluctant to undergo further pertussis nucleic acid testing after their symptoms improved, preventing an assessment of the duration of *Bordetella pertussis* PCR positivity. Lastly, the limited number of patients included in this study restricts the robustness of our conclusions. Future prospective studies with a larger cohort and multi-center design are needed to validate and expand upon our findings.

## Conclusions

In conclusion, our study demonstrated that on-admission real-time PCR Ct values and WBC counts are associated with disease severity in children with pertussis. Patients presenting with low Ct values may require close monitoring and aggressive intervention due to their increased risk of severe disease outcomes.

## Data Availability

The datasets used and/or analyzed during the current study are available from the corresponding author upon reasonable request.

## Abbreviations

COVID-19: Corona Virus Disease 2019
Ct: Cycle threshold
PCR: Polymerase chain reaction
DNA: Deoxyribonucleic acid
DTaP: Diphtheria, Tetanus, Pertussis
WBC: White blood cell
IFV: Influenza virus
RSV: Respiratory syncytial virus
HMPV: Human metapneumovirus
PIV: Parainfluenza virus
HRV: Human rhinovirus
HAdV: Human adenovirus
HCoV: Human coronavirus
HBoV: Human bocavirus
MP: Mycoplasma pneumoniae
LOS: Length of stay
OR: Odds ratio
CI: Confidence interval
PT: Pertussis toxin
